# Thermal and Morphological Properties of Poly(L-Lactic Acid)/Poly(D-Lactic Acid)-B-Polycaprolactone Diblock Copolymer Blends

**DOI:** 10.3390/ma13112550

**Published:** 2020-06-03

**Authors:** Eckhard Weidner, Stephan Kabasci, Rodion Kopitzky, Philip Mörbitz

**Affiliations:** 1 Faculty of Mechanical Engineering, Ruhr University Bochum, 44780 Bochum, Germany; weidner@vtp.rub.de; 2 Fraunhofer UMSICHT, Fraunhofer Institute for Environmental, Safety, and Energy Technology, Osterfelder Str. 3, 46047 Oberhausen, Germany; stephan.kabasci@umsicht.fraunhofer.de (S.K.); rodion.kopitzky@umsicht.fraunhofer.de (R.K.)

**Keywords:** poly(lactic acid), stereocomplex, plasticizer, copolymers, migration

## Abstract

Due to the brittle nature of poly(lactic acid) many attempts have been made to flexibilize this polyester for applications such as thin films and foils. However, due to complex phase behavior, many drawbacks for plasticizer and blend components are described. To overcome miscibility, post crystallization and migration issues a principle of click-chemistry was employed to change the molecular characteristics from external to internal plasticization by fixation of a plastisizing unit with help of a stereocomplex crystallization. Hydroxyl terminated polycaprolactone oligomers were used as a macroinitiator for the ring opening polymerization of d-lactide, resulting in blockcopolymers with plasticizing unit polycaprolactone and compatibilizing poly(d-lactic acid)-blocks. The generated block copolymers were blended with a poly(l-lactic acid)-matrix and formed so called stereocomplex crystals. In comparison to unbound polycaprolactone the polycaprolactone blocks show a lower migration tendency regarding a solution test in toluene. Besides that, trapping the plasticizing units via stereocomplex also improves the efficiency of the plasticizer. In comparison to polymer blends with the same amount of non-bonded polycaprolactone oligomers of the same molecular weight, block copolymers with poly(d-lactic acid) and polycaprolactone can shift the glass transition temperature to lower values. This effect can be explained by the modulated crystallization of the polycaprolactone-blocks trapped into the matrix, so that a higher effective amount can interact with the poly(l-lactic acid)-matrix.

## 1. Introduction

The production of bio-based and biodegradable plastics is one important part of the bio-economy. The use of bio-based plastics saves fossil resources and, in most cases, helps reducing greenhouse gas emissions. A prominent representative of these materials is poly(lactic acid) (PLA). High strength and elastic modulus make this material excellent for rigid applications. However, several drawbacks have to be overcome regarding the use of pure PLA or blends with PLA as major component in flexible thin films. Commercially available PLA in general consists of the l-lactic acid monomer and will thus in the following referred as PLLA, poly(l-lactic acid). Due to the brittle nature of PLLA, many attempts have been made to flexibilize this polyester [1]. However, low molecular weight plasticizers do not provide substantial improvements, primarily due to migration phenomena [2,3]. Another way of toughening PLLA is blending it with other polymeric components, such as polyethylene glycol (PEG) [4] or polycaprolactone (PCL) [5]. However, complex (co-)crystallization, aging and separation effects take place in these systems. To overcome those separation effects, the use of block copolymers is one possible solution. Block copolymers can affect the blend morphology and properties in two different ways. First, as a phase compatibilizer of the immiscible components of the block copolymer reducing interphase tension and increasing the fineness and stability of the dispersed phase (compatibilization), and second as an in-situ-compatibilized blend component itself, thereby increasing solubility and dispersion.

Whereas a phase compatibilizing blockcopolymer is an additive, used in low amounts and modifying the morphology of the blend, the use of block copolymers as compatibilized plasticizer can be seen as a shift from external to internal plasticization. From a thermodynamic point of view, an interface phenomenon such as a low compatibility of two different polymers can be influenced by increasing their interactions. If no covalent bonds exist, the interaction is described by the sum of all attracting forces. This also includes the lattice energy of forming crystals. In order to increase the interactions between PLA block copolymers and a PLA matrix, the use of opposite enantiomeric forms of lactic acid and thus the formation of a stereo complex may be appropriate. Stereocomplex formation between PLLA and poly(d-lactic acid) (PDLA) has been reported by Ikada et al. first [6]. Besides a higher melting point of this crystal configuration the mechanical properties differ from pure enantiomeric PLA. By measuring the mechanical properties of PLLA/PDLA cast films and polarization microscopy images, Tsuji and Ikada have developed a model that explains the changed mechanical properties of the stereocomplex PLA compared to pure PLLA by the morphology of the stereocomplexes [7]. Both spherulites of homo crystals and crystallites of stereocomplexes are connected by tie chains. Due to the smaller size of the stereocomplex crystallites, longer chain segments protrude into the amorphous phase and statistically more tie chains are formed than in homo crystallization. The higher density of tie chains was therefore assumed to explain the increased elongation at break [7]. Similar results were found by Rodriguez et al. [8]. The stereocomplex formation between PLLA and PDLA block copolymers has been reported by Stevels et al. [9]. Li et al. provide an overview of studies on the formation of stereo complexes of PLLA-PDLA block copolymers [10].

The influence of PDLA-PEG-PDLA triblock copolymers on the crystallinity of PLLA has been independently investigated recently by several researchers [11,12,13]. Jing et al. show that the formation of a stereo complex depends on the chainlength of the PDLA block length of the blockcopolymer [11]. An optimum is achieved at a molecular weight of approx. 14,000 g·mol^−1^, where only stereocomplex crystals are formed. Furthermore, they describe the influence of the PEG block length. With an increasing block length of PEG, the melting temperature of the stereocomplex crystals decreases. This effect is explained by the increasing degree of imperfection as the PEG central block length increases [11]. Song et al. demonstrate a reduced ordered state in blends of PLLA and PDLA-PEG-PDLA triblock copolymers by thermal optical analysis [12]. While a blend of PLLA and PDLA exhibits circular spherulites at a temperature of 120 °C, the dendritic forms of blends with PLLA and PDLA-PEG-PDLA triblock copolymers show a less symmetrical shape [12]. On the other hand, the researchers describe an increased crystallization rate of the blends with PLLA and the triblock copolymers compared to blends of PLLA and PDLA measured in isothermal differential scanning calorimetry (DSC). The reason for this effect is the diluting effect of the miscible PEG midblock and thus increasing the chain mobility and rearrangements [12].

Rathi et al. describe similar results. This research group also investigated the properties of blends with PLLA and PDLA-PEG-PDLA triblock copolymers. Thermoanalytical investigations as well as infrared and Raman spectroscopic analyses show that the triblock copolymers form stereo complexes with the PLLA matrix. The elongation at break of the blend with 15% by weight triblock copolymer increases to 72%, while the modulus of elasticity remains approximately constant [13]. Furthermore, the authors compare the morphology of the blends with that of pure PLLA. Polarization microscope images of PLLA and PLLA with 15 w% triblock copolymer clarify the difference. While PLLA crystals grow in large symmetric spherulites, the crystals of PLLA and the PDLA-PEG-PDLA triblock copolymer grow in small crystallites with amorphous space between each crystallite. As Tsuij and Ikada [7] report, the crystallites of the stereocomplex PLA are smaller than the spherulites of the homopolymers and are surrounded by amorphous structures. Consequently, the improved elongation at break of the blend compared to the PLLA homopolymer can be explained by the compatible content of soft PEG and by a difference in morphology between stereocomplexes and homocrystals. In another publication, Rathi et al. compare different PLLA blends with A-B-A block copolymers, where the A blocks consist of PDLA and the B block consists of miscible poly(ethylene glycol-co-propylene glycol) (PEPG) and incompatible poly(ethylene-co-butylene) (PEB) [14]. While blends with a miscible B-block show a high improvement in elongation at break of up to 488%, this is only increased to 26% with an incompatible B-block. The morphology of blends containing miscible triblock copolymer is similar to that of PLLA blends with PDLA-PEG-PDLA. In blends with a incompatible B-block, small spherical phases exist besides crystallites. Raman mapping showed that the amorphous phase in incompatible B-blocks consists exclusively of PLA, whereas in compatible B-blocks, there is also non-crystallized PEPG in the amorphous phase. The authors assume that in this case non-crystalline PEPG and PLA form a continuous amorphous soft phase, which is the cause of the softening of the material.

In this article, linear A-B-block copolymers consisting of a compatibilizing PDLA-block and a partially miscible plasticizing PCL-block were examined as non-migrating plasticizers for PLLA. As the miscibility of PCL and PLLA depends on the molecular weight of the components, defined block lengths of both, the PCL oligomers and the PDLA-blocks were synthesized.

## 2. Materials and Methods

Materials: Poly(l-lactic acid) used in this study is a commercial product of NatureWorks LLC, PLA2003D. Commercial grade d-lactide was supplied by Corbion NV. ε-caprolactone (99.5%, Carl Roth GmbH & Co. KG, Karlsruhe, Germany); toluene (99.5%, Carl Roth GmbH & Co. KG, Karlsruhe, Germany) and 1-octanol (99%, Carl Roth GmbH & Co. KG, Karlsruhe, Germany) were purchased from Carl Roth. The catalyst tin(II)ethylhexanoate (stannous octanoate, 92.5%–100.0%, Sigma-Aldrich Chemie GmbH, Taufkirchen, Germany) was supplied by Sigma Aldrich. All materials were used as supplied.

Synthesis of PCL/ PDLA oligomers and block copolymers: Oligomers of PCL and PDLA were prepared by ring opening polymerization of ε-caprolactone or d-lactide respectively, catalyzed by stannous octanoate. In both cases, the polymerization was initiated by 1-octanol, so that the resulting products are PCL or PDLA oligomers, which are monohydroxyl terminated. First, ε-caprolactone or d-lactide was heated together with the initiator in a 250 mL reaction flask under nitrogen atmosphere to 120 °C oil bath temperature. Then 0.1 mol% tin 2-ethylhexanoate referred to d-lactide was dissolved in approx. 3 mL toluene, added to the reaction mixture, and the oil bath temperature was raised to 130 °C for caprolactone or 145 °C for d-lactide, respectively. The temperature is kept constant for at least 80 min and the reaction mixture is stirred at 100 rpm by means of a driven spreader blade stirrer (RZR 2102 Control, Heidolph Instruments GmbH & Co. KG, Schwabach, Germany). The progress of the reaction was checked by infrared spectroscopy and the reaction time was adjusted if necessary. In order to purify the synthesized PCL oligomers, the products were dissolved in chloroform and precipitated in cold ethanol. PDLA-PCL block copolymers were synthesized and purified analogously to the PDLA oligomers, using mono alcohol terminated PCL as initiator. Therefore, the PCL block lengths are defined by the length of the PCL initiator used and the PDLA block length can be determined by measuring the overall molecular weight of the resulting block copolymer. The naming of the products is based on the molecular weights determined by size exclusion chromatography (SEC), therefore the number in PCL_X stands for the molecular weight of PCL in g·mol^−1^ and PDLA_Y_PCL_X stands for the molecular weights of each block in the block copolymers.

Infrared spectroscopy was employed to monitor the progress of the reaction of d-lactide monomer, using an infrared spectrometer (Vertex 70, Bruker GmbH, Ettlingen, Germany) with Golden Gate ATR (Golden Gate, Specac Ltd., Orpington, UK) For this purpose, reaction samples were taken every 5 min. The conversion was determined by the ratio of the intensity maxima of the IR bands at 935 cm^−1^ (COO deformation vibration in the lactide ring) and 1750 cm^−1^ (CO stretching vibration). From the intensity ratio, the amount of remaining d-lactide was estimated. The intensity ratio of pure d-lactide is defined as 100%.

Size exclusion chromatography: Molecular weights were measured by size exclusion chromatography (SEC) based on a SECurity SEC system from PSS. The system was calibrated with narrowly distributed polymethylmethacrylate standards (PMMA, ReadyCal, PSS GmbH, Mainz, Germany). The solvent used was 1,1,1,3,3,3-hexafluoro-2-propanol (HFIP, 99.9%, ChemPur GmbH, Karlsruhe, Germany). The results shown are based on the evaluation of the measured values of a viscosity (ETA-2010, PSS GmbH, Mainz, Germany) or light scattering detector (PSS SLD 7000, Brookhaven Instrument Inc., Holtsville, NY, USA). The evaluation was done with the software WinGPC^®^UniChrom (8.3, PSS GmbH, Mainz, Germany).

Thermo gravimetric analysis: Thermo gravimetric analyses were carried out in a Netzsch 209 F1 (209 F1, NETZSCH GmbH & Co. Holding KG, Selb, Germany). Approximately 10 mg sample was weighed into an open aluminium crucible. The loss of mass was recorded in a temperature range from 30 °C to 550 °C. The heating rate is 10 K/min. If the difference in decomposition temperatures is sufficiently high, the mass loss of the individual stages can be used to determine the mass fraction of the respective components of the samples.

Preparation of binary blends: In addition to cast films of polymer blends out of a polymer blend solution in chloroform, individual components were mixed in a two-roll mill (LRM-SC-110/T3E, Labtech Scientific Ltd., Embourg, Belgium). Both rolls were operated at different speeds and temperatures. Roller 1 was heated to 180 °C and rotated at 15 rpm. Roller 2 was heated to 160 °C and rotated at 30 rpm. The gap width between the two rotating rolls is 0.28 mm. First of all, PLA2003D granulate was melted. Thereafter, the synthesized block copolymers were slowly added into the melt. The materials were rolled until a rotating melt is created between the rollers. The melt was then drawn off the rolls to repeat the rolling process two more times. The cast films as well as the blends from the roll were used to produce films with a film thickness of approx. 200 µm using a laboratory press. For this purpose, the material to be tested was heated for 1 min without pressure at 210 °C, a pressing force of 100 kN was set (press surface 400 cm^2^), deaerated for 0.1 s and then cooled down under pressure for 1.5 min after 1 min full pressing time at 100 kN.

Differential scanning calorimetry: DSC was performed with a power compensated DSC (DSC 8000, Perkin Elmer, Rodgau-Jügesheim, Germany). For the measurements, approx. 8 mg sample from at least three different points on the films was weighed into an aluminum crucible. In order to ensure the discharge of any gases that may be produced, the lid was provided with a hole in advance. The temperature program for the PLA based blends consists of a first heating up to 220 °C with 20 K/min, then cooling down to 0 °C at a cooling rate of 10 K/min and a second heating to 220 °C at a heating rate of 10 K/min. In order to characterize the PCL oligomers a temperature program consisting of heating up to 100 °C with 20 K/min, then cooling down to −100 °C at a cooling rate of 10 K/min and a second heating to 100 °C at a heating rate of 10 K/min. At the maximum and minimum temperature, the temperature is kept constant for 3 min.

Polarized optical microscopy: Thermo-optical analysis was carried out with an optical microscope (DM2500, Leica, Wetzlar, Germany) in hot stage. The PLLA blends were melted at 220 °C and then cooled to 110 °C crystallization temperature with 75 °C/min.

Migration tests were performed by treating the film materials with toluene, which is a non-solvent for PLLA and a solvent for PCL. For this purpose, pressed films were placed in toluene for 72 h. The films were weighed in advance under normal conditions. After removal of the films from the toluene, the surface toluene was dabbed with a paper towel, the films were dried for 24 h in a vacuum drying oven (VDL 115, Binder GmbH, Tuttlingen, Germany) at 60 °C, 10^−2^ mbar and then weighed again under normal conditions. The weight loss due to this treatment with toluene was determined for evaluation.

Scanning electron microscope: SEM examinations of the films treated with toluene were carried out with a SEM (Vega3, Tescan GmbH, Dortmund, Germany) with an acceleration voltage of 20 kV and a secondary electron detector SE. The foil surfaces were sputtered with gold using a sputter-coater (Cressington 108, Cressington, Dortmund, Germany) with 40 mA for 120 s.

## 3. Results and Discussion

### 3.1. Blends of PLLA and PCL Oligomers

In order to consider the plasticizer potential of the linear PCL oligomers, the miscibility of the respective PCL oligomers in PLLA was calculated theoretically. Based on the group contribution theory, the solubility parameters δ_i_ according to Hildebrand were determined for PCL and PLLA, Table 1.

From the calculated solubility parameters, the Flory-Huggins interaction parameter χ can be derived [17]:

(1) $$\chi =\frac{V}{\mathrm{RT}}{\left(\delta_{1}-\delta_{2}\right)}^{2},$$

which allows the Flory-Huggins miscibility formula [18] to be used to obtain a miscibility curve for different molecular weights of the PCL oligomers.

(2) $$\frac{\Delta G_{m}}{\mathrm{RT}}=\frac{\phi_{a}}{N_{a}}\ln\phi_{a}+\frac{\phi_{b}}{N_{b}}\ln\phi_{b}+\phi_{a}\phi_{b}\cdot \chi ,$$

**Figure 1:**
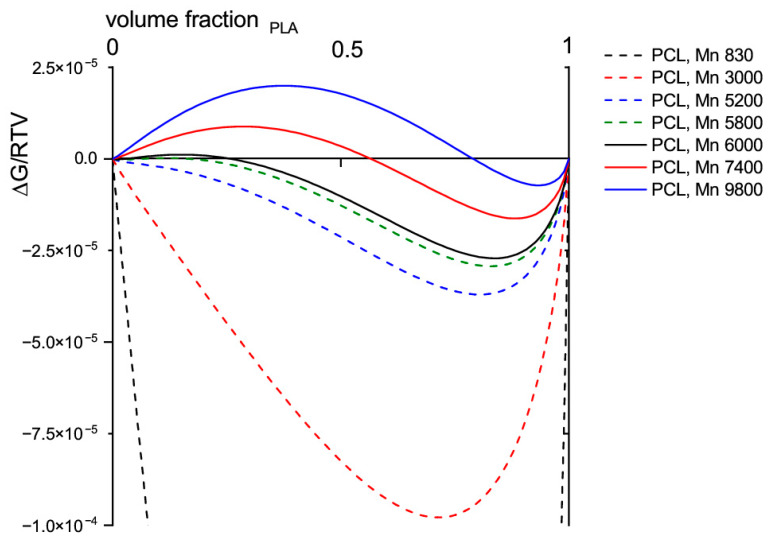
Gibbs free energy of mixing of PLLA (molecular weight M_n_ = 80,000 g·mol^−1^) with PCL oligomers of different molecular weight M_n_.

The molecular weight M_n_ of 80,000 g·mol^−1^ for PLLA, determined by GPC, was used as a basis. Figure 1 shows the calculated Gibbs free mixing energy ΔG_m_ for each molecular weight M_n_ of the PCL oligomers against the volume fraction of PLLA.

The calculation reveals that the mixing behavior of PCL oligomers in a PLLA matrix is strongly dependent on their molecular weight. While oligomers with a molecular weight above g·mol^−1^ are completely incompatible with the PLLA matrix, and oligomers with a molecular weight below 5700 g·mol^−1^ are compatible in all mixing ratios, oligomers with a molecular weight in between are partially compatible. For the following investigations, a PCL mass fraction of 10 w% will be considered. Due to nearly the same densities of PLLA and PCL this mass fraction corresponds to a volume fraction of ~10%. For this volume ratio, PCL oligomers up to a molecular weight of 12,500 g·mol^−1^ are completely miscible in PLLA. Accordingly, all blends considered in this paper should be miscible based on the theoretical calculations.

The theoretical plasticizing effect of compatible PCL oligomers can be described using the Fox equation. As can be seen from the DSC analysis of the PCL oligomers, the glass transition temperature of the investigated oligomers is not dependent on molecular weight and can therefore be considered constant at −59 °C for the calculation of the theoretical plasticizing effect. Such a behavior is unusual, especially in case of shorter molecular chains, since the glass transition temperature is strongly dependent on the molecular weight of the oligomers due to an increased free volume of shorter chains. For PCL, however, this effect has been described by Izuka et al. The research group determined the glass transition temperatures of PCL oligomers with molecular weights of 2000 to 20,000 g·mol^−1^. The deviation of the glass transition temperature was determined to be as small as 3 °C [19]. To calculate the potential plasticizing effect of the PCL oligomers, the glass transition temperature of the PLA/PCL blends is calculated using the weight fractions ω_1_, ω_2_ and the respective glass transition temperatures T_g,1_ and T_g,2_ using Equation (3) developed by Fox [20].

(3) $$\frac{1}{T_{g,\mathrm{mix}}}=\frac{\omega_{1}}{T_{g,1}}+\frac{\omega_{2}}{T_{g,2}}$$

For a PCL mass fraction of 10% by weight, the glass transition temperature of a PLLA-PCL blend is reduced from 58.0 °C to 41.9 °C assuming ideal mixing behavior.

### 3.2. Thermal Properties of Blends of PLLA and PCL Oligomers

**Figure 2:**
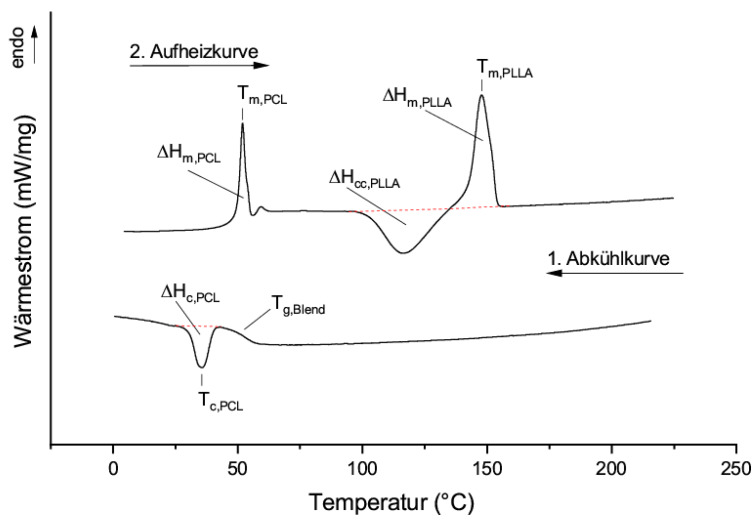
Exemplary differential scanning calorimetry (DSC) curves of the second heating and first cooling cycle of blends of PLLA and PCL oligomers.

The influence of PCL as a blending component for PLA has been extensively described in the literature [5,21,22]. The investigations described below serve to evaluate and understand the efficiency of PCL as a classical plasticizer for PLA. For this purpose, solvent cast films of PLLA and the synthesized PCL oligomers (mass fraction of 10%) were produced and pressed to determine their thermal properties in a DSC analysis. A comparison with theoretical considerations is given to evaluate the plasticizer efficiency. Figure 2 shows a schematic representation of a thermogram of films with 90% PLLA and 10% PCL oligomer by weight. For evaluation, the first cooling run and the second heating cycle are examined.

In the second heating curve a post-crystallization peak ΔH_cc,PLLA_, as well as a melting peak ΔH_m,PLLA_ of PLLA are visible. The melting peak of PCL overlays the glass transition temperature of the blend. The evaluation of the crystallization peaks of PCL, ΔH_C,PCL_, and the glass transition point T_g,Blend_ is therefore performed using the cooling run. Table 2 summarizes the thermal properties of PLLA blends with PCL of different chain lengths. To calculate the actual crystallinity X_C,PLLA_ of the blends, the melting enthalpy is corrected for the post-crystallization enthalpy and divided by the correction factor (1 − ω_PCL_). In this way, only the crystallinity of the PLLA chains is considered:

(4) $$X_{C,\mathrm{PLLA}}=\frac{\Delta H_{m,\mathrm{PLLA}}-\Delta H_{\mathrm{CC},\mathrm{PLLA}}}{\Delta H_{m,\mathrm{PLLA}}^{0}}/\left(1-\omega_{\mathrm{PCL}}\right),$$

ΔH^0^_m_,_PLLA_ = 93.6 J/g [23].

The actual proportion of PCL ω_PCL_ is determined by the weight ratio of PLLA and PCL. The crystallinity of the PCL phases is calculated as described using the crystallization peak from the 1st cooling cycle.

(5) $$X_{C,\mathrm{PCL}}=\frac{\Delta H_{c,\mathrm{PCL}}}{\Delta H_{m,\mathrm{PCL}}^{0}}/\omega_{\mathrm{PCL}},$$

ΔH^0^_m_,_PCL_ = 139.5 J/g [24].

The crystallinity of the PCL phase X_C,PCL_ is dependent on the chain length of the oligomers. After a significant increase of the crystallinity of the PCL phase from PCL_830 to PCL_2200, the crystallinity of the PCL phase in the PCL/PLLA blend stays in a region of ~47%–53%. This behavior cannot be explained on the basis of statistical thermodynamics of polymer crystal formation. From a thermodynamic point of view, the formation of crystals from short polymer chains is preferred. While the degree of order of the crystalline structures and thus the crystal melting temperature of a homopolymer generally decreases as the polymer chain length decreases, the melting enthalpy increases as the polymer chain length decreases [25]. However, the data summarized in Table 2 do not result from measurements on a homopolymer but describe the behavior of a crystallizing PCL phase in a polymer blend. In this case, a thermodynamic competition between the mixing enthalpy and lattice energy of the PCL oligomers in the PLLA blend occurs. Thus, the increased crystallinity of the longer PCL chains can be attributed to their reduced miscibility.

From the same dataset (Table 2), a correlation between the molecular weight of the PCL in the PLLA and the glass transition temperature of the blend can be seen. Despite the theoretical miscibility of all examined blends, the glass transition temperature of blends with short PCL_830 oligomers is lower compared to blends with longer PCL oligomers. Furthermore, the theoretical glass transition temperature of 41.9 °C, calculated by the Fox-Flory equation, is not reached for any of the examined blends of PLLA and 10 w% PCL. The measured reduced plasticizing efficiency of the PCL oligomers and the dependence of the glass transition temperature on the molecular weight of these can be explained by two physicochemical effects:

- The miscibility of the individual components
- The crystallinity of the individual components

According to Flory-Huggins, smaller oligomers are more miscible than longer molecular chains. They are thus better distributed in the spaces between the polymers, can create more effective volume or free volume in more places, and thus have a plasticizing effect. In addition, long-chain molecules may have an increased occurrence of crystalline phases. Crystalline phases cannot interact with amorphous regions of other polymers. Accordingly, the crystalline fractions cannot act as classical plasticizers and are less efficient with regard to the shift in the glass transition temperature. Due to the measured shifts of the glass transition temperature and the melting peaks of PCL phases it is deduced, that the crystallization affinity of PCL results in a separation of PLLA and PCL phases and thus, in less efficient plastizicing. This hypothesis can be supported by a theoretical calculation of the plasticizing effect of the amorphous PCL phases. Except for the PCL oligomer with the shortest chain length of 830 g·mol^−1^, the crystallinity of the PCL differs from 47.3% to 53.0%. Consequently, only 4.8% to 5.3% amorphous PCL do effectively act as plasticizer in the PLLA/PCL blend. According to the fox equitation, this amount of actually plasticizing PCL would lead to a shift of the glass transition temperature from 58 °C to approximately 50 °C, which represents the measured glass transition temperatures of the blends very well.

### 3.3. Thermal Properties of Blends of PLLA and PDLA-PCL Diblock Copolymers

Blends of PLLA and PDLA-PCL-diblock copolymers were made by two roll mixing process. The amount of each copolymer in the blend was chosen to give a PCL amount of ~10 w% in the blend based on thermos gravimetric analysis of the diblock copolymers. In Table 3 the weight ratios of PCL and PDLA of each block copolymer calculated from thermo gravimetric analysis are summarized.

**Figure 3:**
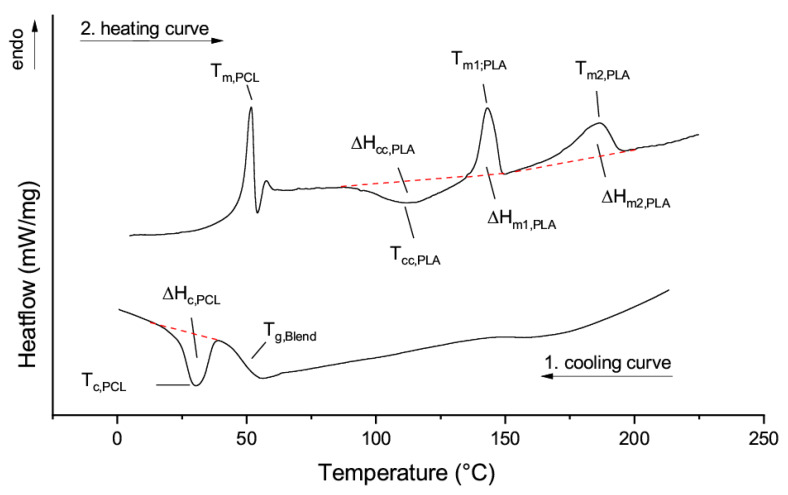
DSC analysis of the blends PLLA and PDLA-b-PCL block copolymers, left: top: 2nd heating run, bottom left: 1st cooling run.

In the non-isothermal DSC, similar transitions can be seen as in the blends with PCL oligomers. The glass transition temperature of the blend T_g_ and the crystallization peak Tc of the PCL phase are evaluated using the cooling run as described in Section 3.1. A significant difference can be seen in the second heating rate. In addition to the glass transition of PLLA overlaid by the melting of the semi-crystalline PCL phase T_m,PCL_, the cold crystallization of the amorphous PLLA phase T_CC,PLA_, and the melting of the PLLA homocrystals T_m1,PLA_, a further endothermic peak at elevated temperatures can be recognized: T_m2,PLA_. This peak can be attributed to the melting of stereo-complex crystals [26]. Figure 3 shows an exemplary thermogram of the cooling run and the second heating of a PLLA matrix blended with PDLA_2400_PCL_5800.

For the evaluation and discussion of the effects of the PDLA-PCL block copolymers on the thermal behavior of the blends, the crystallinity of the blends is described first. The results of the non-isothermal DSC are summarized in Table 4. The crystallinity X_C,PLA_ is also calculated here using a modification of Equation (4) that includes the melting enthalpies of both PLA crystal forms, the one of the homocrystal of PLLA ∆H_m1_ and that of the stereocomplex ∆H_m2_. For calculating the standard melting enthalpy of the mixed PLA crystals ∆H^0^_m,mix_, theoretical standard melting enthalpies for PLLA homocrystals and stereocomplex crystals must be taken into account.

(6) $$X_{c,\mathrm{PLA}}=\frac{\Delta H_{m1}+\Delta H_{m2}-\Delta H_{\mathrm{CC}}}{\Delta H_{m,\mathrm{mix}}^{0}}/\left(1-\omega_{\mathrm{PCL}}\right),$$

The theoretical maximum standard melting enthalpy of the blends ∆H^0^_m,mix_ is determined from the relative proportions of possible stereocomplex crystals X_SC_ and homocrystals X_HC_ and their standard melting enthalpies ∆H^0^_m1_ and ∆H^0^_m2_, Equation (7).

(7) $$\Delta H_{m,\mathrm{mix}}^{0}=\Delta H_{m1}^{0}\cdot X_{\mathrm{HC}}+\Delta H_{m2}^{0}\cdot X_{\mathrm{SC}},$$

The standard melting enthalpies ∆H^0^_m1_ and ∆H^0^_m2_ are known from the literature [23]. For homocrystals this is 93.6 J/g and for stereocomplexes 142 J/g. The relative proportions of possible stereocomplexes and homocrystals X_S_ and X_H_ are determined by the ratio of PLLA and PDLA components. For this purpose, the PDLA fraction in the resulting blend X_PDLA_ is calculated from the amount of each copolymer ω_Copolymer, Blend_ and the weight ratio of PDLA in each copolymer ω_PDLA, Copolymer_, Equation (8).

(8) $$X_{\mathrm{PDLA}}=\omega_{\mathrm{Copolymer},\;\mathrm{Blend}}\cdot \omega_{\mathrm{PDLA},\;\mathrm{Copolymer}},$$

This PDLA fraction in the blend X_PDLA_ can theoretically form stereocomplex crystals with the same amount of PLLA, resulting in the fraction of stereocomplex X_SC_ calculated from Equation (9).

(9) $$X_{\mathrm{SC}}=2\cdot X_{\mathrm{PDLA}},$$

The theoretical proportion of homocrystals X_HC_ is described by the amount of PLLA not forming stereo complexes. Therefore, it is derived from the total amount of PLLA and PDLA in the blend (held constant at 90 w%, since the PCL fraction is fixed at 10 w%) substracted by the theoretical amount of stereocomplex crystals X_SC_, Equation (10).

(10) $$X_{\mathrm{HC}}=0.9-X_{\mathrm{SC}},$$

The crystallinity of the PCL phase in the polymer blend is calculated using Equation (5).

It can be seen that a second melting peak at higher temperatures occurs when the PDLA block length is larger than 2400 g·mol^−1^, which is due to the melting of stereocomplex crystals. In general, the higher the melting point of a crystal form, the more thermodynamically stable it is. This thermodynamic stability depends on disorder in the crystal structure. Disorders in the frozen state of the crystals can be caused by chain ends and in the case under consideration by disturbances caused by the PCL blocks. In the case of the stereo complex crystals, the highest melting point of 205 °C occurs with the PLLA blend with the block copolymer PDLA_7000_PCL_3000. This block copolymer has the largest PDLA block length of 7000 g·mol^−1^. Therefore, the effect of the chain ends on the crystal structure is less pronounced, since their number is comparatively small. The blend with the block copolymer PDLA_2400_PCL_5800 shows the lowest melting point of the stereocomplex crystals at 186 °C. Here, the long PCL block leads to disorder in the crystal and thus reduces its melting point. Additionally, it can be noticed that the total crystallinity of all PLA components in this blend is only slightly increased compared to the blends without stereocomplex formation. This is caused by the fact that there is almost no nucleating effect of the stereocomplex crystals on the crystallization of the PLLA-phase. Such an effect occurs for PDLA molecular weights of more than 3500 g·mol^−1^. Blends with copolymers having a PDLA block length above 3500 g·mol^−1^ show a significant increase of the overall crystallinity.

**Figure 4:**
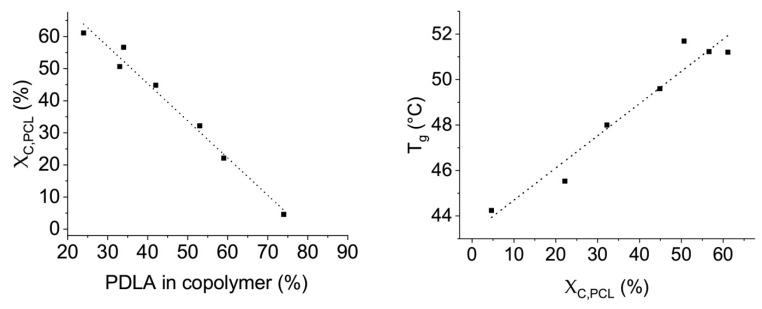
Dependence of the crystallinity of the PCL phase on the PCL fraction in the block copolymer (**left**) and the glass transition temperature of the PLLA phase on the crystallinity of the PCL phase (**right**). The drawn straight lines represent the results of linear regression calculations.

Interestingly, there are further thermal properties of the blends that depend on the nucleation by the PDLA blocks. As in the case of blends of PLLA and PCL oligomers, crystallinity of the PCL phases can be observed in blends with PDLA-b-PCL block copolymers. For blends, in which the block copolymer has a sufficiently large PCL block for crystallization (>2200 g·mol^−1^), and the PDLA block is larger than 2400 g·mol^−1^ so that stereocomplexes with the PLLA matrix occur, the PCL crystallinity values only reach half of the PCL crystallinity in PLA blends with PCL oligomers. The formation of a stereocomplex of the PDLA-PCL block copolymers leads to a reduction in the crystallinity of the PCL phase. Accordingly, a correlation between the fraction of PDLA in the block copolymer and the crystallinity of the PCL phase in the blend can be derived, Figure 4 left. A higher proportion of the PDLA in the block copolymers leads to a lower crystallinity of the PCL phase. Furthermore, the reduced PCL crystallinity results in higher shifts in the glass transition temperature of PLLA and thus in an increased plasticizing efficiency of the additives, Figure 4 right. This significant shift of glass transition temperatures to lower temperatures in the case of blends with PDLA-PCL block copolymers consisting of medium PCL block length supports the hypothesis that a PDLA block allows PCL oligomers to be anchored in a PLLA matrix via stereocomplex formation and thus to have a more efficient plasticizing effect. For longer PCL block lengths, especially when combined with shorter PDLA blocks (<2400 g·mol^−1^), the crystallization behavior of the PCL phase thermodynamically dominates the blend structure.

**Figure 5:**
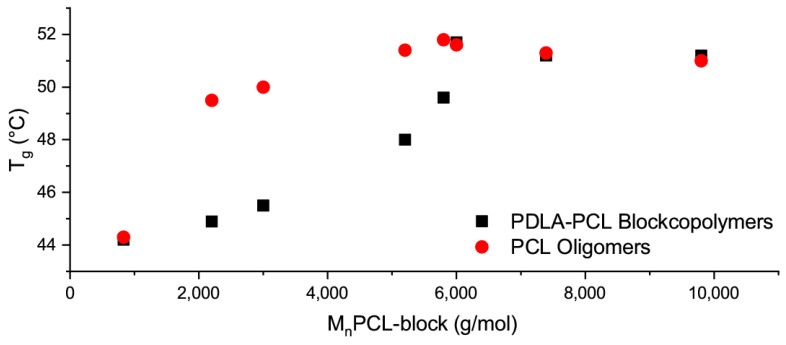
Dependence of glass transition temperature on molecular weight of PCL chains for blends of PLLA with PCL oligomers (red) and PLLA and PDLA-b-PCL block copolymers (black).

The smaller the crystalline portion of the PCL phases, the more amorphous PCL can interact with the PLLA matrix, and the more the glass transition temperature shifts towards lower temperatures. Compared to the plasticizing effect of PCL oligomers, this shift in the glass transition temperature is higher for block copolymers with PDLA molecular weight of over 3500 g·mol^−1^, Figure 5.

### 3.4. Thermooptical Properties of Blends of PLLA and PDLA-b-PCL Diblock Copolymers

**Figure 6:**
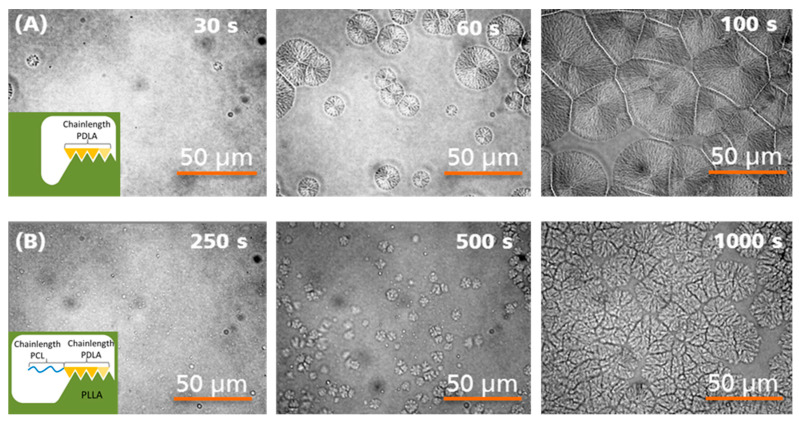
Thermo-optical analysis of blends from PLLA with (**A**) PDLA_5000 and (**B**) PDLA_5000_PCL_830.

It is known that the addition of PDLA oligomers can nucleate PLLA and accelerate crystal growth [7]. Consequently, block copolymers of PDLA and PCL should also have a nucleating effect on PLLA when a stereocomplex is formed. Therefore, thermo-optical analyses were performed on thin films of blends of PLLA and PDLA-PCL block copolymers to investigate their crystallization behavior. For comparison, the crystallization of blends of PLLA with PDLA oligomers with the same block length as in the block copolymer was additionally observed. The thin films were melted at a temperature of 220 °C, cooled down to 120 °C and the films were kept constant at that temperature for at least 30 min. Under the selected conditions, crystallization could be observed in thermo-optical analysis for blends with block copolymers whose PDLA block length is 3800 g·mol^−1^ or greater. Figure 6 shows typical crystal formations for the blend with (A) PDLA_5000 oligomer and (B) PDLA_5000-b-PCL_830 block copolymer.

A significant difference in the shape of the crystal structures between PDLA-b-PCL nucleated and PDLA nucleated PLLA is clearly visible in Figure 6. While blends of PLLA with PDLA oligomers form round symmetrical, densely grown spherulites (Figure 6, Series A, top row), the crystallites of the blends with PDLA-b-PCL are less symmetrical (Figure 6, Series B, bottom row). In addition, the crystallites of the latter blends are smaller and have gaps within the crystal structures. The model established by Jing et al. can explain these differences in the degree of ordering of the crystalline structures [11]. Related to the findings in our research the PCL blocks adherent to the PDLA increase the distance between the single growing crystals, so that the degree of order of spherulith structures decreases and gaps within the crystal structure occur. Furthermore, the PCL blocks also decrease the crystal growth rate of the optically visible crystallites, Figure 6. Basically, however, PDLA-PCL block copolymers with a PDLA block length of 3800 g·mol^−1^ or greater exhibit a nucleating effect on a PLLA matrix. This is another indication that the crystallization of the PDLA blocks with the PLLA matrix anchors the PCL blocks into it.

### 3.5. Migration of PCL Oligomers and PDLA-b-PCL Copolymers in Blends with PLLA

**Figure 7:**
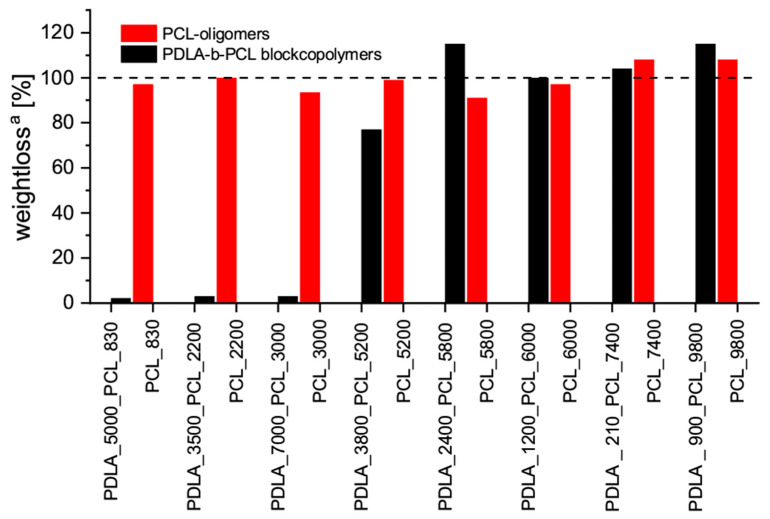
Migration behavior of the blends of PLLA and PDLA-b-PCL block copolymers (black) and PCL oligomers (red) in toluene; ^a^ weightloss in % based on the additive amount of PDLA-PCL block copolymers or PCL oligomers used.

Apart from thermal and optical properties of the examined blends, the migration ability of the PDLA-PCL block copolymers was determined by the solvent resistance of the blend and compared to the resistance of blends with PCL oligomers. The weightloss of pressed films stored in toluene for 72 h was measured, Figure 7.

The analysis of the data shows that treatment with toluene allowed 100% migration of the PCL oligomers out of the sample for all investigated PLLA-PCL blends. In some cases, more additive leaks from the matrix than theoretically possible. A control experiment, in which pure PLLA was treated with toluene showed no loss of mass under these test conditions. It is therefore assumed that these deviations are due to measurement inaccuracies caused by contaminations or inhomogeneity of the film. Nevertheless, a significant difference is observed when comparing PLLA blends with PCL oligomers and those with PDLA-b-PCL block copolymers. Again, the blends in which the molecular weight of the PDLA segment in the block copolymer is higher than 3500 g·mol^−1^ show a changed behavior. In these cases, the loss of mass due to toluene treatment is significantly lower. For blends with block copolymers in which the PCL block additionally has a molecular weight of less than 3000 g·mol^−1^, the loss in mass is less than 5%. These results show that PCL block copolymers which are bound to a PLLA matrix via a stereo complex show a reduced migration in initially good solvents. This effect can be explained by the strong interaction between PDLA block and PLLA matrix in the stereocomplex crystal. For homopolymer blends of PLLA and PDLA the reduced solubility in good PLA solvents has already been described [7]. However, our research clarifies that this behavior is not limited to PLLA /PDLA homopolymer blends, and that PCL plasticzing units can be bound to the matrix by the formation of an insoluble stereocomplex crystal.

**Figure 8:**
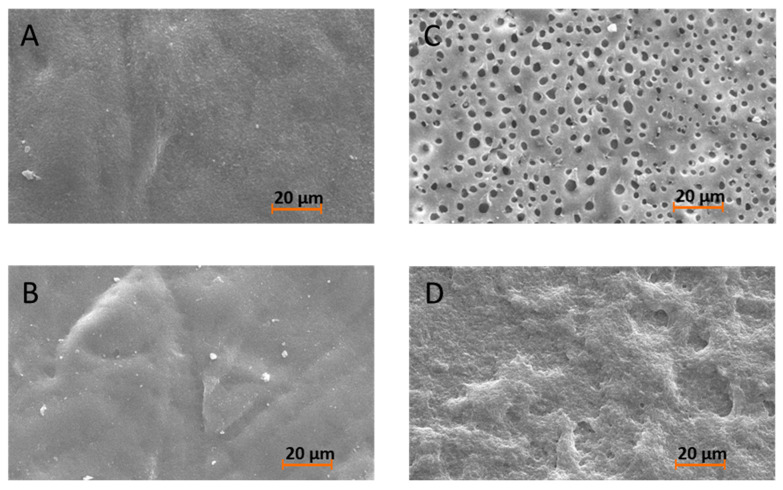
SEM images of the film surfaces of the blends of PLLA and PDLA-b-PCL block copolymers: (**A**): PDLA_3500_PCL_2200, (**B**): PDLA_5000_PCL_830 and blends of PLLA and PCL oligomers (**C**): PCL_2200, (**D**): PCL_830 after toluene treatment.

To support the results of the gravimetric analysis of the migration of PCL oligomers into toluene, the surfaces of the film materials after treatment with toluene were examined using SEM micrographs. Figure 8 shows the surface of the PLLA blends with PDLA-PCL block copolymers (Figure 8A,B) and PCL oligomers (Figure 8C,D) after toluene treatment.

The images show a smooth surface for blends with pronounced stereocomplex formation, i.e., PDLA_3500_PCL_2200 (Figure 8A) and PDLA_5000_PCL_830 (Figure 8B), even after treatment with toluene. In the case of blends where the gravimetric analysis already indicated migration into toluene, surface defects (roughness, holes of extracted PCL oligomer) can be seen in the SEM images. This behavior shows again that in blends with PCL oligomers a migration of the PCL phases occurs, while the bound PCL blocks remain in the film material. In addition, the morphology of blends with linear PCL oligomers can be estimated by these SEM images. As described above the phase segregation of PLLA and PCL oligomers is determined by their molecular weight. Thermal investigations of the blends of PLLA and oligomeric PCL show that a separate PCl crystalline phase occurs when its molecular weight is above 830 g mol^−1^. The SEM images of the toluene-treated PLLA blends confirm this phase behavior. While in the SEM image of PLLA blend with PCL_2200 (Figure 8C) small round holes are clearly visible, in case of the blend with PCL_830 (Figure 8D) only a few holes are implied. These findings confirm the thesis, that in blends with PCL_2200 small separated PCL droplets emerge and in case of PCL_830 the oligomers are soluble in the PLLA matrix. Compared to the surface of the bound PCL block copolymers (Figure 8A,B) the surface of the toluene treated film of PLLA blend with PCL_830 (Figure 8D) is less plain. Therefore, it is assumed that the unbound PCL oligomers migrate into the toluene regardless of their solubility. However, the stereocomplex anchored PCL-blocks do not show any migration in the SEM images and thus this analysis confirms the results of the gravimetric analysis.

## 4. Conclusions

The results of this study show that anchoring of partly incompatible PCL-blocks into PLLA via stereocomplex crystallization is possible. The thermal and morphological properties of the blends of PLLA and PDLA-PCL diblock copolymers can be controlled by adjusting the chain lengths of both blocks. Block copolymers with a compatibilizing PDLA block of 2400 g·mol^−1^ and longer show a second melting peak in DSC analysis, which corresponds to the formation of stereocomplex crystals. This formation of stereocomplex crystals between the PDLA blocks and the PLLA matrix leads to a modulated crystallinity of the PCL blocks. The formation of stereocomplex results in lower crystallization tendency of the PCL blocks and thus in a more efficient plasticization of the PDLA-b-PCL compared to PCL oligomers.

Furthermore, the formation of stereocomplex and the associated strong anchoring of the PCL blocks in the PLLA matrix results in a lower migration tendency of the plasticizing PCL units. Films consisting of PLLA and block copolymers with long PDLA blocks and short PCL blocks show a higher resistance to a toluene-treatment than films of PLLA and PCL oligomers of the same molecular weight. The results of the gravimetric tests are supported by SEM images of the toluene treated films. PLA blends with anchored PCL blocks can thus be an adequate possibility for the use in flexible film applications. The characterization of mechanical properties of PLLA blends with blockcopolymers consisting of a compatibilizing block of PDLA with defined plasticizing blocks will be investigated on extruded thin films. The puplication of these results is planned for the near future in a continuative article. Furthermore, the concept of trapping a component in PLLA via stereocomplex can be expanded to other applications.

## Figures and Tables

**Table 1:** Hildebrand solubility parameters **δ** of PCL/ PLLA calculated by group additivity method [15,16,17].

	δ_Small_	δ_Hoy_	δ_vanKrevele*n*_	δ_Ø_	T_g_^1^
PCL	19.08	19.45	18.29	18.94	−59.0
PLLA	19.86	20.55	18.86	19.75	58.0

^1^ measured by DSC.

**Table 2:** Thermal properties of blends of PLLA containing 10 w% of PCL oligomers of different chain lengths.

	$\Delta {H_{\mathrm{cc}\;\mathrm{PLLA}\;}}^{a}$ (J/g)	T_cc PLLA_^a^ (°C)	$\Delta {H_{m1,\;\mathrm{PLLA}\;}}^{a}$ (J/g)	T_m1, PLLA_^a^ (°C)	X_C,PLLA_^a^ (%)	$\Delta {H_{c,\;\mathrm{PCL}\;}}^{b}$ (J/g)	T_c, PCL_^b^ (°C)	X_C,PCL_^b^ (%)	T_g, Blend_^b^ (°C)	T_g,PCL Oligomer_^c^ (°C)
PLLA	20.0	124.7	20.0	147.1	0	-	-	-	58,0	-
PCL_830	25.7	105.6	25.8	139.8	0.1	0.2	18.0	1.4	44.3	−59.0
PCL_2200	1.3	120.6	2.1	145.0	0.8	6.6	12.3	47.3	50.4	−58.7
PCL_3000	10.1	118.0	11.7	144.3	1.5	6.7	23.4	48.0	50.9	−58.5
PCL_5200	21.0	116.4	21..5	147.8	0.5	6.8	35.7	48.7	51.4	−59.6
PCL_5800	20.1	115.9	21.0	147.5	0.9	6.7	34.5	48.0	51.8	−59.0
PCL_6000	3.8	118.1	4.8	144.1	1.0	6.9	35.2	49.5	51.6	−59.3
PCL_7400	1.1	121.4	2.0	145.1	0.9	7.2	33.3	51.6	51.9	−59.8
PCL_9800	8.4	117.5	10.2	144.4	1.7	7.4	22.3	53.0	51.3	−59.4

^a^ determined from 2nd heating run; ^b^ determined from 1st cooling run.; expectation 10% PCL 41.9 °C; ^c^ determined in a separated DSC measurement.

**Table 3:** Data of thermogravimetric analysis of the PDLA-PCL diblock copolymers.

PLLA Blend with	ω_PDLA, Copolymer_ (%)	ω_PCL, Copolymer_ (%)	ω_Copolymer, Blend_ (%)
PDLA5000_PCL830	72	28	31
PDLA3500_PCL2200	55	45	24
PDLA7000_PCL3000	59	41	25
PDLA3800_PCL5200	47	53	19
PDLA2400_PCL5800	41	59	15
PDLA1200_PCL6000	18	82	12
PDLA210_PCL7400	24	76	15
PDLA900_PCL9800	20	80	13

**Table 4:** Thermal properties of blends of PLLA and PDLA-PCL block copolymers.

PLLA Blend with	ΔH_cc,PLLA_^a^ (J/g)	ΔH_m1, PLLA_^a^ (J/g)	T_m1,PLLA_^a^ (°C)	ΔH_m2, PLAsc_^a^ (J/g)	T_m2,PLAsc_^a^ (°C)	ΔH_m_^0^_, mix_ (J/g)	X_C,PLA_^a^ (%)	ΔH_m,PCL_^b^ (J/g)	X_C,PCL_^b^ (%)	T_g,Blend_^b^ (°C)
PDLA5000_PCL830	7.0	0.5	142.4	17.4	193	105.8	10.3	0.2	1.4	44.2
PDLA3500_	2.5	6.6	147.0	27.3	195	97.0	32.4	3.1	22.2	45.8
PCL2200										
PDLA7000_	10.1	9.8	143.5	24.7	205	98.5	24.8	3.5	25.1	45.5
PCL3000										
PDLA3800_	10.1	10.7	142.7	15.9	194	92.9	17.8	4.0	28.7	48.0
PCL5200										
PDLA2400_	4.9	4.9	143.0	3.5	186	90.2	3.9	5.7	40.9	49.6
PCL5800										
PDLA1200_	-	0.5	146.0	-	-	86.3	0.5	6.4	45.9	51.1
PCL6000										
PDLA210_PCL7400	-	0.5	147.0	-	-	87.7	0.5	9.8	70.3	51.2
PDLA900_PCL9800	3.7	3.8	147.0	-	-	86.8	0.1	7.1	50.9	51.2

^a^ determined from 2nd heating run, ^b^ determined from 1st cooling run.
